# Validation and reliability of the Malaysian English version of the psychosocial impact of dental aesthetics questionnaire for adolescents

**DOI:** 10.1186/s12955-017-0632-x

**Published:** 2017-03-21

**Authors:** Wan Nurazreena Wan Hassan, Zamros Yuzadi Mohd Yusof, Mohd Zambri Mohamed Makhbul, Siti Safuraa Zahirah Shahidan, Siti Farhana Mohd Ali, Rashidah Burhanudin, Maria Jirom Gere

**Affiliations:** 1 Department of Paediatric Dentistry and Orthodontics, Faculty of Dentistry, University of Malaya, Kuala Lumpur, 50603 Malaysia; 2 Department of Community Oral Health and Clinical Prevention, Faculty of Dentistry, University of Malaya, Kuala Lumpur, Malaysia; 3 Orthodontic Unit, Klinik Pergigian Cahaya Suria, Ministry of Health Malaysia, Kuala Lumpur, Malaysia; 4 Faculty of Dentistry, University of Malaya, Kuala Lumpur, Malaysia

**Keywords:** Malocclusion, Oral health-related quality of life, Adolescent, Cross-cultural adaptation, Impact-related need, Patient-reported outcome measure

## Abstract

**Background:**

The Malay version of the Psychosocial Impact of Dental Aesthetics Questionnaire has been validated for use by Malaysian adolescents. Although Malay is their national language, English is widely used as the lingua franca among Malaysians of different ethnicities. This study aimed to validate an English version of the PIDAQ adapted for use by Malaysian adolescents to optimize data capture from adolescents who prefer English as the medium for communication.

**Methods:**

The published English version of PIDAQ was pilot tested on 12- to 17-year-old adolescents, resulting in a few modifications to suit the Malaysian variety of English. Psychometric properties were tested on 393 adolescents who attended orthodontic practices and selected schools. Malocclusion was assessed using the Malocclusion Index, an aggregation of Perception of Occlusion Scale and the Aesthetic Component of the Index of Orthodontic Treatment Need, by the subjects (MI-S) and investigators (MI-D). Data were analysed for internal consistency and age-associated invariance, discriminant, construct and criterion validities, reproducibility and floor and ceiling effects using AMOS v.20 and SPSS v.20.

**Results:**

The item *Don’t like own teeth on video* of the Aesthetic Concern (AC) subscale was not relevant to a large proportion of participants (11.7%). Therefore, it was removed and the Malaysian English PIDAQ was analysed based on 22 items instead of 23 items. Confirmatory factor analysis showed good fit statistics (comparative fit index: 0.902, root-mean-square error of approximation: 0.066). Internal consistency was good for the Dental Self-Confidence, Social Impact and Psychological Impact subscales (Cronbach’s alpha: 0.70-0.95) but lower (0.52–0.62) though acceptable for the AC subscale as it consisted of only 2 items. The reproducibility test was acceptable (intra-class correlations: 0.53–0.78). For all PIDAQ subscales, the MI-S and MI-D scores of those with severe malocclusion differed significantly from those with no or slight malocclusion. There were significant associations between the PIDAQ subscales with ranking of perceived dental appearance, need for braces and impact of malocclusion on daily activities. There were no floor or ceiling effects.

**Conclusion:**

The adapted Malaysian English PIDAQ demonstrated adequate psychometric properties that are valid and reliable for assessment of psychological impacts of dental aesthetics among Malaysian adolescents.

## Background

Oral health is no longer seen exclusively as an absence of oral diseases. Good oral health is considered to be having or maintaining optimal functional, social and psychological well-being [1]. Therefore, researchers have developed a number of oral health-related quality of life (OHRQoL) instruments to measure clinically relevant outcomes from the patient’s perspective, which are used to measure general oral health needs or to measure specific diseases or conditions such as the impact of malocclusion on their well-being.

Like their counterparts in other countries, many adolescents in Malaysia desire to undertake orthodontic treatment [2]. Thus, instruments that could measure the impact of the malocclusion on the adolescent’s OHRQoL would provide more objective information on the perceived needs of the adolescents. This may support treatment priority as the impact of the malocclusion on patients’ OHRQoL is considered on top of clinical oral health treatment measures.

The Psychosocial Impact of Dental Aesthetics Questionnaire (PIDAQ) is an instrument that has been developed specifically for measuring impacts related to dental aesthetics and arrangement [3]. It was improvised from previous scales [4–7] that were able to associate subjects’ malocclusion with perception of their dental aesthetics [3] with few added items to form 4 subscales of 23 items measuring the impact of malocclusion on the dental self-confidence (DSC), social impact (SI), psychological impact (PI) and aesthetic concern (AC). The DSC is a 6-item subscale that measures the impact of dental appearance on a positive self-concept. The other 3 subscales are negative domains: SI has 8 items that measure anxiety levels about other people’s reaction when the subjects expose their teeth, PI has 6 items that measure negative emotions about the dental aesthetics, and AC has 3 items that measure displeasure with the image of the subject’s teeth in different conditions. The questionnaire has shown good psychometric properties in cross-cultural adaptations in adults [8–12] as well as in adolescents [13–15]. The instrument has recently been cross-culturally adapted into the Malay language and has been shown to be valid and reliable for use by Malaysian adolescents [16].

In order for the instrument to operate well, it must be validated to be used for the target population. In Malaysia, Malay (also referred to as *Bahasa Malaysia*, or Malaysian language) is the national language. However, English is widely used as a lingua franca among urban Malaysians. Clinical impressions suggest that communication between a clinician and an adolescent patient is commonly in the language that the patient prefers, usually either in Malay or in English. It was also noted that a good number of adolescents especially in the urban areas prefer to express themselves in the lingua franca of English regardless of their background. Exposure of English has become more widespread as it is regularly used in Malaysian media including the internet. Thus, a Malaysian English version of the PIDAQ as an alternative to the Malay PIDAQ is necessary and may greatly benefit OHRQoL studies to capture the psychometric information of adolescents who may not be comfortable to answer Malay-medium instruments. This will prevent exclusion of subjects due to language barrier. The aim of this study was to develop and test a Malaysian English version of the PIDAQ instrument for use by Malaysian adolescents and to evaluate its psychometric properties.

## Methods

The study comprised both linguistic and psychometric validations. The adapted English version was intended to be provided as an alternative to the Malay PIDAQ for subjects who are less proficient in the Malay language or who prefer to use English. Therefore, the process of linguistic validation was parallel to that used for the linguistic validation process of the Malay PIDAQ, monitored closely to ensure that the conceptual, item and semantic equivalences did not differ from the Malay version. None of the authors were familiar with German, the language used for development and validation of the original [3] and adolescent [15] versions of the PIDAQ. Therefore, the professionally translated, published English versions [3, 15] were the basis for the Malaysian versions. Linguistic validation involved face and content validations by experts in orthodontics (WNWH and MZMM) and in dental public health and OHRQoL measures for children (ZYMY), pre-testing, comparison with the Malay PIDAQ and final review of the draft English PIDAQ.

Following content validation by the experts, the published English version for adolescents was pilot tested on seven patients aged 12–14 and on seven patients aged 15–17 non-randomly selected from the orthodontic waiting list. The pilot test was conducted at the Faculty of Dentistry, University of Malaya by the investigator (WNWH). The adolescents were asked to answer the questionnaire independently and the time taken to complete the questionnaire was recorded. Next, a discussion with the participants was conducted paying attention to the participants’ feedback on the general layout of the questionnaire including the instructions, questionnaire items and answer options. In the discussion, some words that were considered ambiguous were discussed and replaced with suitable words as suggested by the participants. They were encouraged to suggest words that they would find more suitable to their level of comprehension. When words that could not be understood by the adolescents could not be replaced with other suitable words, the published original version was consulted, tested verbally and discussed to see if subjects understood the meaning. Examples of words that were deemed difficult by the participants were *content, reveal, fear, funny looks, self-conscious, upset, envy* and *ashamed*. Participants also agreed that the answer option *not relevant* should be included for the item *Don’t like own teeth on video* for those who had never seen a video recording of themselves.

Following the pilot test, a meeting was held between two of the authors, one an orthodontist (WNWH) and the other an expert in OHRQoL measures (ZYMM); both were native Malay speakers and proficient in both English and Malay languages. The aim of the meeting was to discuss the outcomes of the pilot test and compare with the back translation of the Malay PIDAQ before agreeing on the draft adapted English PIDAQ. During the discussion, close attention was given to the content and wording of the adapted English PIDAQ to ensure that conceptual and item equivalence were achieved between the original instrument and the adapted English PIDAQ. Conceptual equivalence was assessed to ensure the questions reflect the same concept and the concepts are meaningful to the targeted cultures and languages. Item equivalence was achieved when the meaning of each item was maintained during the adaptation process [17].

Next, the psychometric properties of the adapted English PIDAQ were tested on non-randomly selected adolescents who had not been involved in the pilot study. Sample size calculation was done to detect mis-specified factor loadings comparing the two age-groups, using A-priori Sample Size Calculator for Structural Equation Models [18]. Given 4 PIDAQ subscales with 23 items, the recommended sample size for each age group at a power level of 0.80 and a probability level of 0.05 for model structure was 166 [19, 20]. This concurred with the rules-of-thumb of 4 to 10 subjects per variable [21]. Convenience sampling was done, which included participants who volunteered from 4 schools in the northern part of peninsular Malaysia and participants in the orthodontic waiting lists from 2 orthodontic government clinics in Kuala Lumpur. To account for variation in developmental changes in adolescents [15], participants were divided into two narrow age groups: The lower secondary school children 12 to 14 years old (Forms 1 and 2) and upper secondary school children 15 to 17 years old (Forms 3 to 5). For completeness of analysis, the participants were also analysed collectively. Due to the policy of the Ministry of Education Malaysia that does not permit involvement of students who will take the national examinations in that year, i.e., the Form 3 and Form 5 classes of secondary school, participants from schools were recruited from those in Forms 1, 2 and 4. Participants from the orthodontic clinics comprised 12- to 17- year-olds who requested orthodontic treatment. Exclusion criteria included those who were having or have had orthodontic treatment and those with craniofacial deformities. The questionnaire was self-administered. Participants completed the questionnaire either in the classroom or in the orthodontic clinic waiting area. For the re-test, questionnaires were redistributed to 25% of the participants after 2 weeks.

Responses were scored from 1 to 5 on a 5-point Likert scale: *not at all* (score 1), *a little* (score 2), *somewhat* (score 3), *strongly* (score 4) and *very strongly* (score 5). For each subscale, scores were tabulated from the total item scores. Total scores of the subscales SI, PI and AC and the reversed scores of the positive domain DSC were summed to provide the total PIDAQ score, which is a measure of the impact of the dental aesthetics on the psychosocial well-being of patients [22]. Low PIDAQ scores would indicate a low impact of dental aesthetics on OHRQoL while high scores would indicate high negative psychosocial impact.

Data were analysed using IBM-SPSS-AMOS v.20 and IBM-SPSS-Statistics v.20. Chi-squared test and Fisher exact test were done to compare equality of the proportions of the demographics between age-groups. Internal consistency was measured by confirmatory factor analysis (CFA) and Cronbach’s α for each subscale. The CFA calculated estimates of the maximum likelihood discrepancy. Goodness of fit of the observed data to the model was measured on a comparative fit index (CFI) ≥ 0.90 and root-mean-square error of estimation (RMSEA) < 0.08 [15]. Multiple group comparison was done to determine measurement invariance between the two age groups. Three stages of invariances where the models were further constrained at each stage were tested: In the configural (baseline) model, all free parameters were estimated separately in each group; in the measurement weights model, the paths of the factor loadings were constrained equally across groups; and in the structural covariances model, the estimated factor loadings, factor covariances and factor variances were constrained [23]. Non-invariance across age groups was assessed as ∆CFI ≥ 0.01 when compared against the baseline configural model [24]. Subscales with Cronbach’s α of between 0.70 and 0.95 were also considered to have good internal consistency [25].

The PIDAQ was developed to assess need for treatment in patients requesting orthodontic treatment [17] and to measure orthodontic-specific OHRQoL outcomes [15]. As in the previous study [15], discriminant validity was tested by comparing the relationship of the PIDAQ subscales with perceived need for orthodontic treatment based on the Malocclusion Index [15], which comprised the Aesthetic Component of the Index of Orthodontic Treatment Need (IOTN-AC) and the awareness component of the Perception of Occlusion Scale (POS). The IOTN-AC was rated using a black and white photographic 10-point-scale showing teeth with increasing severity of malocclusion [26]. The POS component comprised 6 items of malocclusion traits [27] and participants were required to evaluate their level of agreement with each item on a 5-point Likert scale from *not at all* to *very strongly*. The self-rated and investigator-rated Malocclusion Indices (MI-S and MI-D, respectively) were adapted for analysis of the severity of malocclusion where the scores of the IOTN-AC and total scores of the POS were standardized, summed up and divided by 2 to give an index value with a 0 mean value [15]. The judgments of six investigators (WNWH, SSFS, SFMA, MZMM, RB and MJG) were calibrated for the MI-D. The inter-operator intraclass correlation (ICC) at T1 was excellent [28] at 0.97 (95% CI = 0.95 to 0.98; *p* < 0.001). Intra-operator ICC scores were also excellent at above 0.75 (*p* < 0.001) and ranged from 0.85 (95% CI = 0.71 to 0.92) to 0.95 (95% CI = 0.90 to 0.97).

The construct validity of the adapted English PIDAQ was tested by comparing the relationship of the PIDAQ subscales with other measures measuring related constructs, i.e., rank of perceived dental appearance and need for braces. For criterion validity test, this was assessed by comparing the relationship of the PIDAQ subscales with perceived impact of malocclusion on daily activities using the Child-Oral Impacts on Daily Performances (Child-OIDP) index [29]. The rank of perceived dental appearance was rated from *Excellent, Good, Average* and *Poor* while the need for braces was rated as *Yes, No* and *Don’t know*. The Child-OIDP is used as a condition-specific (CS) instrument to measure the impacts of malocclusion on daily activities if impacts were attributed to the *Spaces between* and *Position of the teeth* [30]. The third CS item, which was *Deformity of the mouth and face* was excluded as it was not relevant due to the exclusion criteria. The performance score was tabulated by multiplying the frequency (scale from 1 to 3) and magnitude (scale from 1 to 3) of the impact that was attributed to any of the 8 daily activities, i.e., cleaning teeth, eating, emotional stability, smiling, speaking, relaxing, doing schoolwork and socialising. The instrument total score was tabulated by summing up the 8 performance scores. It was scored as 0 if there was no impact on the 8 daily activities. The range of scores for each performance was 0–9 and the index was 0–72.

An independent *t*-test was applied to compare the relationship between the PIDAQ subscales and total PIDAQ scores with the malocclusion index (MI-S/MI-D) scores of those with no or slight malocclusion (lower quartiles) and severe malocclusion (upper quartiles). The effect size was tabulated as 2 t/√df where t is the *t* test value and df is the degree of freedom [31]. Kruskal-Wallis and Mann-Whitney statistics were used to assess the relationship between PIDAQ and the other subjective measures mentioned. The Pearson correlation coefficient was calculated to assess the relationship between the PIDAQ and CS-Child-OIDP total performance scores of the eight daily activities. The CS-Child-OIDP total performance scores were tabulated only when the impact was due to malocclusion.

In terms of reproducibility test, the standard error of measurement (SEM) was calculated as the square root of the residual variance of the ANOVA analysis, and the smallest detectable change (SDC) was calculated as 1.96 × √2 × SEM [25, 32]. The paired *t*-test determined if there was any significant change in PIDAQ subscales of informants between the first and second tests. Limits of agreement were calculated as mean change ± 1.96 × standard deviation of the changes [33]. The ICC for absolute agreement by two-way random effects models was calculated [25].

Floor and ceiling effects within each subscale were calculated as the percentage of the achieved lowest and highest possible scores. Floor or ceiling effects were considered present if the prevalence was more than 15% [25].

## Results

In total, 393 participants responded (12–14 year old age group = 203, 15–17 year old age group = 190). Table 1 shows selected demographics of the participants. Frequencies across the demographics showed no statistically significant differences between the age groups.

**Table 1:** Demographics of the participants

Demographics	12–14 years (N)	15–17 years (N)	*p*-value
Gender			
Male	81	79	0.76
Female	122	111	
Venue			
Orthodontic Clinics	55	46	0.76
Schools	148	144	
Ethnic			
Malay	163	142	0.56^c^
Chinese	24	31	
Indian	14	15	
Others	2	2	
^a^Severity of malocclusion (Self-rated)			
Lower quartile (No or slight)	48	53	0.50
Middle quartile (Moderate)	103	86	
Upper quartile (Severe)	49	49	
^b^Severity of malocclusion (Investigator-rated)			
Lower quartile (No or slight)	48	46	0.91
Middle quartile (Moderate)	103	99	
Upper quartile (Severe)	52	45	

^a^Self-rated Malocclusion Index (MI-S); ^b^Investigator-rated Malocclusion Index (MI-D); ^c^Fisher exact-test

Initial analysis showed that 11.7% (*n* = 46) of the participants chose *Not relevant* for the item *Don’t like own teeth on video.* This demonstrated that a relatively large proportion of the participants found this item irrelevant to their circumstances. Therefore, based on the recommendation by Jokovic et al*.* [34] and through discussions among the authors, it was decided to remove this item from the AC subscale of the PIDAQ. Thus, the psychometric analysis in this study was based on the shortened version of the Malaysian English PIDAQ that comprised 22 items instead of 23 items.

**Fig. 1:**
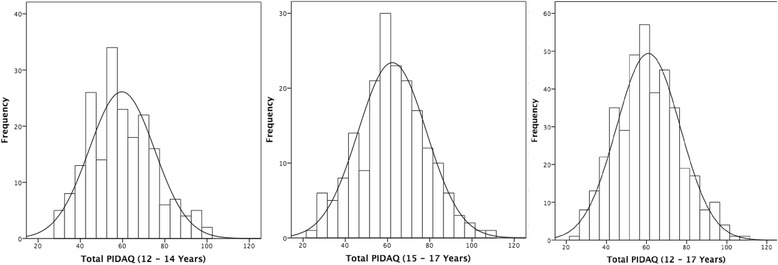
Distribution of the total PIDAQ scores of the participants

In this study, the histogram demonstrated that the data were normally distributed. Thus, the results were described in means and standard deviations (Fig. 1). The mean PIDAQ score was 59.7 (SD = 15.5; Min = 30; Max = 100) for the younger age group, 62.3 (SD = 16.2; Min = 24; Max = 110) for the older age group and 60.9 (SD = 15.9; Min = 24; Max = 110) for the overall population. Independent *t*-test showed no significant difference in mean PIDAQ scores between the younger and older age groups (*p* = 0.10; 95% CI = −5.8 to 0.5).

The factor analysis (Table 2) shows good-fit statistics: the CFI score of Model A was at 0.90, while the RMSEA was less than 0.08, with a small confidence interval. The factor loadings were within acceptable range although one item had a factor loading of less than 0.50. Multi-group invariance test showed that the baseline configural model constrained for age group was 0.898. The ∆CFI was 0.004 (i.e., ∆CFI < 0.01). The CFI of the measurement weights model was 0.893 and structural covariance model was 0.893 (i.e., ∆CFI < 0.01 against the baseline model), confirming invariance across age groups.

**Table 2:** Multi-group confirmatory factor analysis showing the standardised parameter estimates and fit indices

	Model A	Model B	
N	393	203	190
Age group	12–17 years	12–14 years	15–17 years
Fit Indices			
Comparative Fit Index	0.902	0.898	
Root Mean Square Error of Approximation (90% CI)	0.066 (0.060–0.073)	0.048 (0.043–0.053)	
Items in Brief			
Dental Self-Confidence			
• Proud of own teeth	0.78	0.73	0.83
• Like to show their teeth	0.59	0.55	0.63
• Pleased to see own teeth in mirror	0.78	0.82	0.74
• Teeth look nice to others	0.59	0.55	0.62
• Satisfied with own teeth’s appearance	0.63	0.68	0.56
• Find own teeth nice	0.72	0.70	0.74
Social Impact			
• Hold back their smile	0.57	0.58	0.56
• What others think	0.62	0.50	0.74
• Teasing	0.70	0.74	0.67
• People look strange at my teeth	0.66	0.67	0.66
• Shy because of own teeth	0.66	0.68	0.63
• Hiding own teeth	0.65	0.67	0.64
• Stupid comments from others	0.58	0.49	0.67
• Boys/girls find own teeth ugly	0.67	0.63	0.70
Psychological Impact			
• Envy others for their teeth	0.55	0.48	0.59
• Distressed because of others’ nice teeth	0.80	0.76	0.85
• Unhappy about own teeth	0.76	0.77	0.76
• Others have nicer teeth	0.59	0.53	0.66
• Feel bad about own teeth	0.71	0.68	0.73
• Wish to look better	0.44	0.41	0.48
Aesthetic Concern			
• Don’t like own teeth in mirror	0.67	0.63	0.73
• Don’t like own teeth on photos	0.59	0.57	0.61

Table 3 shows the results of internal consistency analyses of the subscales, scale statistics and inter-item correlations of the subscales. The subscales of DSC, SI and PI satisfactorily achieved the Cronbach’s α values of between 0.70 and 0.95 for all age groups. However, the subscales of the AC component was moderately satisfactory, ranging between 0.52 and 0.62, for all age groups. None of the inter-item correlations were ≥ 0.90 for all subscales or ≤0.30 for the DSC and AC subscales. For the SI subscale, the items with inter-item correlations below 0.30 were: between *Hold back their smile* and *What others think* (12–14 years = 0.23), *Shy because of own teeth* (15–17 years = 0.28), *Stupid comments from others* (12–14 years = 0.17; all ages = 0.25) and *Boys/girls find own teeth ugly* (all ages = 0.29); and between *What others think* and (12–14 years = 0.21) and *Stupid comments from others* (12–14 years = 0.20). For the PI subscale, the item *Wish to look better* had inter-item correlations below 0.30 with *Unhappy about own teeth* (12–14 years = 0.23; all ages = 0.29) and *Feel bad about own teeth* (12–14 years = 0.25). None of the item total correlations scores were < 0.30 in all subscales and age groups.

**Table 3:** Internal consistency, scale statistics and inter-item correlations of the subscales

	N	Cronbach’s α	Scale statistics		Inter-item correlations			Item total correlation	
			M	SD	M	Min	Max	Min	Max
12–14 years									
Dental Self-Confidence	202^a^	0.83	15.99	5.56	0.45	0.33	0.62	0.48	0.72
Social Impact	202^a^	0.83	18.54	6.56	0.38	0.17	0.55	0.41	0.66
Psychological Impact	202^a^	0.79	16.44	5.25	0.39	0.23	0.57	0.42	0.65
Aesthetic Concern	203	0.52	4.58	3.58	0.36	0.36	0.36	0.36	0.36
15–17 years									
Dental Self-Confidence	190	0.84	15.21	5.29	0.47	0.36	0.64	0.53	0.73
Social Impact	189^a^	0.86	19.63	6.78	0.43	0.27	0.60	0.47	0.68
Psychological Impact	189^a^	0.83	17.11	5.56	0.46	0.32	0.69	0.49	0.76
Aesthetic Concern	190	0.62	4.74	3.30	0.45	0.45	0.45	0.45	0.45
12–17 years									
Dental Self-Confidence	392^a^	0.84	15.57	5.44	0.46	0.36	0.63	0.54	0.70
Social Impact	391^a^	0.85	19.07	6.69	0.41	0.25	0.51	0.48	0.65
Psychological Impact	391^a^	0.81	16.76	5.41	0.43	0.29	0.63	0.45	0.70
Aesthetic Concern	393	0.56	4.66	1.86	0.47	0.40	0.55	0.48	0.60

^a^Listwise deletion of missing data (i.e., entire observation of individuals omitted when any of the variables were missing)

Table 4 shows the results of discriminant validity analyses of the adapted English PIDAQ. There were statistically significant differences in mean scores between adolescents who rated themselves (MI-S) with no or slight malocclusion and those with severe malocclusions for all subscales and total PIDAQ, and for all age groups (*p* < 0.01). There were statistically significant differences in mean scores between adolescents who were rated by the investigators (MI-D) with no or slight malocclusion and those with severe malocclusions for all subscales in the older and overall age groups and the total PIDAQ scores for the older age group (*p* < 0.01). However, the differences were not statistically significant (*p* > 0.05) in the younger age group for the SI, PI and AC subscales and for total PIDAQ in the younger and overall age groups. In all three age groups, comparison with MI-S and MI-D showed that DSC scores reduced with increasing severity of the malocclusion. In contrast, SI, PI, AC and total PIDAQ (except in comparison to the MI-D in the younger age group) scores increased with increasing severity of malocclusion.

**Table 4:** Discriminant validity of the adapted English PIDAQ with regards to self-rated (MI-S) and interviewer-rated (MI-D) malocclusion

Self-rated Malocclusion (MI-S)												
Age group	12–14 years				15–17 years				12–17 years old			
Quartile (Self perceived malocclusion level)	Lower (Slight)	Upper (Severe)	Effect size	*p* value	Lower (Slight)	Upper (Severe)	Effect size	*p* value	Lower (Slight)	Upper (Severe)	Effect size	*p* value
n	51	50			53	48			101	98		
	Mean (SD)	Mean (SD)			Mean (SD)	Mean (SD)			Mean (SD)	Mean (SD)		
Dental Self-Confidence	19.0 (4.6)	13.2 (5.0)	−1.23	0.00*	18.4 (4.8)	13.2 (4.6)	−1.12	0.00*	18.7 (4.7)	13.1 (4.9)	−1.18	0.00*
Social Impact	14.7 (5.3)	22.8 (6.8)	1.38	0.00*	16.0 (5.9)	23.1 (6.2)	1.19	0.00*	15.4 (5.7)	22.9 (6.5)	1.24	0.00*
Psychological Impact	13.1 (4.5)	19.44 (5.65)	1.28	0.00*	13.3 (4.6)	20.8 (4.00)	1.76	0.00*	13.33 (4.54)	20.2 (5.0)	1.44	0.00*
Aesthetic Concern	3.5 (1.5)	5.9 (2.0)	1.33	0.00*	3.6 (1.5)	5.9 (2.0)	1.31	0.00*	3.6 (1.5)	5.9 (2.0)	1.35	0.00*
Total PIDAQ	50.4 (10.4)	61.3 (12.4)	0.96	0.00*	51.4 (11.0)	63.0 (10.1)	1.12	0.00*	51.1 (10.7)	62.1 (11.3)	1.01	0.00*
Investigator-rated Malocclusion (MI-D)												
Age group	12–14 years				15–17 years				12–17 years			
Quartile (Investigator-rated malocclusion level)	Lower (Slight)	Upper (Severe)	Effect size	*p* value	Lower (Slight)	Upper (Severe)	Effect size	*p* value	Lower (Slight)	Upper (Severe)	Effect size	*p* value
n	48	50			46	47			94	97		
	Mean (SD)	Mean (SD)			Mean (SD)	Mean (SD)			Mean (SD)	Mean (SD)		
Dental Self-Confidence	17.5 (4.9)	13.2 (4.8)	−0.90	0.00*	17.1 (5.3)	13.3 (5.1)	−0.75	0.00*	17.3 (5.1)	13.2 (4.9)	−0.83	0.00*
Social Impact	16.9 (6.3)	19.2 (7.2)	0.34	0.10	17.3 (6.7)	21.0 (6.8)	0.56	0.01*	17.1 (6.5)	20.0 (7.1)	0.43	0.00*
Psychological Impact	15.4 (5.2)	16.5 (5.5)	0.22	0.29	14.6 (5.5)	18.5 (5.3)	0.73	0.00*	15.0 (5.3)	17.4 (5.5)	0.45	0.00*
Aesthetic Concern	4.2 (1.8)	4.8 (1.8)	0.31	0.11	4.2 (1.7)	5.4 (1.8)	0.73	0.00*	4.2 (1.8)	5.1 (1.8)	0.49	0.00*
Total PIDAQ	54.0 (10.9)	53.6 (12.6)	−0.03	0.89	53.1 (11.7)	58.2 (1.7)	0.44	0.04*	53.5 (11.2)	55.6 (12.2)	0.18	0.22

*p < 0.05

In terms of construct validity test analyses, rank of the participants’ perceived dental appearance showed that there were statistically significant associations between all PIDAQ subscales and total PIDAQ scores with self-rated rank of their dental appearance (*p* < 0.01) for all age groups (Table 5). For DSC subscale, mean scores gradually lowered as the participants rated their teeth from excellent to poor. The trend was statistically significant for all age groups. Conversely, mean scores were gradually increased in SI, PI, and AC subscales, and total PIDAQ scores, respectively, as the participants rated their teeth from excellent to poor. The trend was statistically significant for all age groups.

**Table 5:** Construct validity of the adapted English PIDAQ with regards to self-perceived dental appearance rank

PIDAQ variables	Rate Appearance	N	PIDAQ Scores					*p* value
			Mean	SD	Quartiles			
					Lower	Middle	Upper	
12–14 years								
Dental Self-Confidence	Excellent	20	20.0	5.3	14.0	22.5	23.8	0.00*
	Good	111	17.6	4.9	14.0	18.0	20.0	
	Average	54	12.7	4.4	9.8	13.0	16.0	
	Poor	17	9.9	4.2	7.0	8.0	11.5	
Social Impact	Excellent	20	16.7	5.1	12.0	16.0	20.5	0.00*
	Good	111	17.1	5.8	12.0	16.0	22.0	
	Average	54	19.7	6.5	14.8	19.0	24.5	
	Poor	17	25.7	7.0	21.0	27.0	30.5	
Psychological Impact	Excellent	20	14.8	5.2	10.5	15.0	17.0	0.00*
	Good	111	15.1	4.7	12.0	15.0	19.0	
	Average	54	17.9	4.7	14.0	17.5	21.3	
	Poor	17	21.7	5.5	18.0	23.0	26.0	
Aesthetic Concern	Excellent	20	3.8	1.7	2.0	3.0	5.8	0.00*
	Good	111	4.1	1.9	3.0	4.0	5.0	
	Average	54	5.1	1.9	4.0	5.0	6.0	
	Poor	17	6.5	2.2	4.5	6.0	8.5	
Total PIDAQ	Excellent	20	55.2	8.7	49.5	53.5	60.5	0.02*
	Good	111	54.0	10.9	46.0	54.0	62.0	
	Average	54	55.4	11.2	46.8	53.0	66.3	
	Poor	17	63.8	12.1	53.0	67.0	74.0	
15–17 years								
Dental Self-Confidence	Excellent	18	19.9	6.5	13.8	21.0	25.5	0.00*
	Good	78	17.5	4.4	14.0	17.0	21.0	
	Average	73	13.3	3.8	10.5	13.0	16.0	
	Poor	21	9.2	2.6	6.5	10.0	11.5	
Social Impact	Excellent	18	17.7	6.9	9.8	18.0	22.8	0.00*
	Good	78	16.8	5.7	12.8	17.0	20.0	
	Average	73	21.6	5.6	17.5	22.0	26.0	
	Poor	21	25.6	8.1	19.0	25.0	31.5	
Psychological Impact	Excellent	18	15.4	5.9	9.8	15.5	20.0	0.00*
	Good	78	14.3	4.7	11.0	13.0	18.0	
	Average	73	19.1	4.4	15.0	20.0	22.5	
	Poor	21	22.2	5.5	17.5	24.0	27.0	
Aesthetic Concern	Excellent	18	4.0	2.3	2.0	3.0	6.0	0.00*
	Good	78	4.1	1.6	2.8	4.0	5.0	
	Average	73	5.3	1.4	4.0	5.0	6.0	
	Poor	21	6.1	2.3	4.0	6.0	8.0	
Total PIDAQ	Excellent	18	56.6	11.3	45.0	54.5	64.8	0.00*
	Good	78	52.6	11.0	44.0	53.0	61.3	
	Average	73	59.3	10.5	52.0	59.0	67.5	
	Poor	21	63.1	14.1	50.0	67.0	74.0	
12–17 years								
Dental Self-Confidence	Excellent	38	20.0	5.8	14.0	21.5	24.3	0.00*
	Good	189	17.6	4.7	14.0	18.0	20.5	
	Average	127	13.0	4.0	10.0	13.0	16.0	
	Poor	38	9.5	3.4	7.0	9.0	11.3	
Social Impact	Excellent	38	16.9	6.0	11.8	16.0	22.0	0.00*
	Good	189	17.0	5.7	12.5	17.0	21.0	
	Average	127	20.8	6.0	16.0	20.0	26.0	
	Poor	38	25.6	7.5	19.8	27.0	31.0	
Psychological Impact	Excellent	38	15.1	5.5	10.0	15.0	18.3	0.00*
	Good	189	14.8	4.7	11.0	15.0	18.0	
	Average	127	18.6	4.6	15.0	19.0	22.0	
	Poor	38	22.0	5.4	17.8	23.5	26.3	
Aesthetic Concern	Excellent	38	3.9	2.0	2.0	3.0	6.0	0.00*
	Good	189	4.1	1.6	3.0	4.0	5.0	
	Average	127	5.2	1.6	4.0	5.0	6.0	
	Poor	38	6.3	2.2	4.0	6.0	8.0	
Total PIDAQ	Excellent	38	55.8	9.9	47.8	55.0	63.3	0.00*
	Good	189	53.4	11.0	45.0	54.0	61.5	
	Average	127	57.6	10.9	50.0	56.0	57.0	
	Poor	38	63.4	13.0	50.0	67.0	74.0	

*p < 0.05

There were also statistically significant associations between the self-perceived need for braces with all PIDAQ subscales and total PIDAQ scores in all age groups (Table 6). Those who felt they needed braces had significantly lower DSC mean scores and significantly higher SI, PI, AC and total PIDAQ mean scores compared to those who felt they did not need braces in all age groups.

**Table 6:** Construct validity of English PIDAQ with regards to self-perceived need for dental correction

PIDAQ variables	Need Braces	N	PIDAQ Scores					*p* value
			Mean	SD	Quartiles			
					Lower	Middle	Upper	
12–14 years								
Dental Self-Confidence	No	68	18.8	4.8	16.0	19.0	22.0	0.00*
	Yes	85	13.3	5.3	9.0	13.0	17.0	
Social Impact	No	68	15.8	5.3	12.0	15.0	19.0	0.00*
	Yes	85	21.1	6.9	15.5	20.0	27.0	
Psychological Impact	No	68	14.7	4.6	11.0	14.0	17.0	0.00*
	Yes	85	18.3	5.6	14.0	17.0	23.5	
Aesthetic Concern	No	68	4.0	1.7	2.3	4.0	5.0	0.00*
	Yes	85	5.3	2.0	4.0	5.0	6.0	
Total PIDAQ	No	68	53.3	10.0	46.0	53.0	58.0	0.01*
	Yes	85	58.0	11.8	49.0	57.0	69.0	
15–17 years								
Dental Self-Confidence	No	47	18.9	4.3	16.0	18.0	22.0	0.00*
	Yes	98	13.4	5.3	10.0	13.0	16.0	
Social Impact	No	47	15.6	5.5	10.0	17.0	20.0	0.00*
	Yes	98	21.7	6.5	17.0	22.0	25.0	
Psychological Impact	No	47	13.3	4.4	9.0	13.0	17.0	0.00*
	Yes	98	19.4	5.1	15.0	20.0	23.0	
Aesthetic Concern	No	47	3.7	1.4	2.0	4.0	5.0	0.00*
	Yes	98	5.4	1.8	4.0	5.0	6.3	
Total PIDAQ	No	47	51.4	9.9	44.0	51.0	57.0	0.00*
	Yes	98	59.9	11.7	51.0	61.0	68.3	
12–17 years								
Dental Self-Confidence	No	115	18.8	4.6	16.0	19.0	22.0	0.00*
	Yes	183	13.4	5.1	10.0	13.0	16.0	
Social Impact	No	115	15.7	5.4	11.0	15.0	19.0	0.00*
	Yes	183	21.4	6.7	16.0	21.0	26.0	
Psychological Impact	No	115	14.1	4.5	10.0	14.0	17.0	0.00*
	Yes	183	18.9	5.3	15.0	19.0	23.0	
Aesthetic Concern	No	115	3.9	1.6	2.0	4.0	5.0	0.00*
	Yes	183	5.3	1.9	4.0	5.0	6.0	
Total PIDAQ	No	115	52.5	10.0	45.0	53.0	58.0	0.00*
	Yes	183	59.0	11.7	50.0	59.0	69.0	

*p < 0.05; Excluded those who answered ‘don’t know’

Table 7 shows the associations between total PIDAQ scores and the prevalence of CSOIDP for all age groups. Those with CSOIDP had significantly higher total PIDAQ mean scores than did those who did not report any impact on their daily activities related to the CSOIDP in all age groups (*p* < 0.001).

**Table 7:** Criterion validity of the adapted English PIDAQ with regards to impact on daily activities attributed to malocclusion

	Mann-Whitney								Pearson correlation	
PIDAQ variables	CSOIDP Prevalence	N	PIDAQ Scores					*p* value	CSOIDP Performance score	*p* value
			Mean	SD	Quartiles					
					Lower	Middle	Upper			
12–14 years		203								
Total PIDAQ	No	163	53.9	10.7	46.0	53.0	61.0	0.00*	0.294	0.00*
	Yes	40	61.8	11.8	52.3	64.5	71.0			
15–17 years		190								
Total PIDAQ	No	134	54.2	11.3	45.0	55.0	63.0	0.00*	0.350	0.00*
	Yes	56	62.6	10.6	54.3	63.0	70.0			
12–17 years		393								
Total PIDAQ	No	297	54.0	11.0	45.0	53.0	62.5	0.00*	0.326	0.00*
	Yes	96	62.2	11.1	54.0	63.5	71.0			

*p < 0.05

The Pearson correlation coefficient showed statistically significant moderate associations between the total PIDAQ scores and the CSOIDP performance scores (Table 7). The association was positively correlated for all age groups.

Table 8 shows the reproducibility test analyses of the adapted English PIDAQ. The ICCs were above 0.70 for the DSC subscale for all groups and for the SI for the younger age group. The ICCs were generally moderate for the rest of the subscales in all age groups, with the lowest ICC score of 0.53 for the SI subscale in the 15–17 years age group. There were no statistically significant differences between the first and second test administrations in all subscales for all age groups.

**Table 8:** Tests of reproducibility for the adapted English PIDAQ

	ICC_agreement_			Paired *t*-test	Bland and Altman		
	(95% CI)	SEM	SDC	M_Diff_ (SD)	Limits of Agreement		
					Lower	Upper	% Within limits
12–14 years (*N* = 53)							
Dental Self-Confidence	0.75 (0.57–0.86)*	3.63	10.06	0.25 (5.18)	−9.90	10.39	62.3
Social Impact	0.78 (0.61–0.87)*	4.46	12.36	0.59 (6.31)	−11.77	12.94	100.0
Psychological Impact	0.68 (0.46–0.82)*	3.80	10.53	0.94 (5.37)	−9.59	11.47	100.0
Aesthetic Concern	0.68 (0.45–0.82)*	1.50	4.15	−0.15 (2.12)	−4.30	4.00	94.3
15–17 years (*N* = 46)							
Dental Self-Confidence	0.82 (0.68–0.90)*	3.63	10.06	−0.28 (4.21)	−8.53	7.97	63.0
Social Impact	0.53 (0.15–0.74)*	5.91	16.39	0.48 (8.36)	−15.91	16.87	100.0
Psychological Impact	0.64 (0.34–0.80)*	4.52	12.53	−0.09 (6.39)	−12.61	12.44	89.1
Aesthetic Concern	0.56 (0.19–0.75)*	1.66	4.6	−0.24 (2.35)	−4.84	4.37	84.8
12–17 years (*N* = 99)							
Dental Self-Confidence	0.78 (0.67–0.85)*	3.35	9.28	0.00 (4.74)	−9.28	9.28	77.0
Social Impact	0.68 (0.52–0.78)*	5.16	14.30	0.54 (7.30)	−13.76	14.83	94.8
Psychological Impact	0.67 (0.51–0.78)*	4.14	11.48	0.47 (5.86)	−11.02	11.95	100.0
Aesthetic Concern	0.62 (0.44–0.75)*	1.57	4.34	−0.19 (2.22)	−4.54	4.15	92.9

*p < 0.05. *CI* confidence interval, *SEM* standard error of measurement, *SDC* smallest detectable change, *M*_*Diff*_ mean differences, *SD* standard deviation

Neither floor nor ceiling effects were detected. The prevalence of the lowest or highest scores for all subscales were below the cut-off value of 15% in all age groups (Table 9). The AC subscale had the highest prevalence of the percentage lowest scores of between 13.7 to 13.8%.

**Table 9:** Floor and ceiling effects of the English PIDAQ

	12–14 years (*N* = 203)		15–17 years (*N* = 190)		12–17 years (*N* = 393)	
	% Floor	% Ceiling	% Floor	% Ceiling	% Floor	% Ceiling
Dental Self-Confidence	3.9	0.5	4.2	0	4.1	0.3
Social Impact	1.0	0	4.7	0.5	2.8	0.3
Psychological Impact	2.5	0	1.6	0.5	2.0	0.3
Aesthetic Concern	13.8	1.5	13.7	1.1	13.7	1.3

## Discussion

The development of the PIDAQ instruments may take years to ensure the validity of the outcome that they purport to measure. Application of these instruments for populations for which they were not originally designed must be revalidated in some form of cross-cultural adaptation process due to the differences in the language and cultural background. Cross-cultural adaptation overcomes the time- consuming process of developing a new measure, taking advantage of the conceptual ability of the instrument to describe and evaluate health status, and allowing comparisons internationally between cultures. Herdman et al*.* [35] described 6 steps that should be taken into account during cross cultural validation: Conceptual equivalence, item equivalence, semantic equivalence, operational equivalence, measurement equivalence and functional equivalence.

Conceptually, the 4 domains of the PIDAQ have been analysed during the cross-cultural adaptation of the Malay PIDAQ [16]. It was found that the 4 domains of the Malay PIDAQ were as relevant to the Malaysian adolescents as the original PIDAQ had been relevant to the adolescents in Germany [15]. During the development of the adapted English PIDAQ, item and semantic equivalences were closely monitored to be as close as possible to the Malay PIDAQ. This would allow for both instruments to be deployed independently or concurrently in a bilingual format. Consequently, the operational equivalence was also found to be similar to the Malay PIDAQ. The questionnaire format, mode of administration and measuring methods were similar to the Malay PIDAQ while the instruction only differed in translation, i.e., the instructions were in the language of the instruments.

During the pilot tests, the format and instructions were found to be acceptable to the participants. In terms of response mode, the item *Don’t like own teeth on video* was not relevant to a few of the participants in the pilot test. As a result, a *not relevant* answer option was added to the item. During the psychometric analyses, a high proportion of participants (11.7%) chose the *not relevant* option indicating the item was not common in the Malaysian setting. Based on the literature, several ways have been recommended to deal with responses that are not within a Likert scale including to exclude subjects with such responses, using adjusted scores or to drop the item [34]. Following discussions with all authors and to prevent errors in future studies involving the instrument, it was decided to have the item removed from the AC subscale. In terms of mode of administration, the Malaysian English PIDAQ was found to be suitable for self-administration – an important consideration since that it has been intended for large sample population study in schools or for participants to answer in the waiting rooms while waiting for their orthodontic appointment. Opportunities for other modes of administration, e.g., verbal expression, were limited during the pilot test discussion. The self-administered mode was considered feasible in Malaysia as the basic literacy rate for English among secondary school children was 93.2% [36], only slightly lower than the basic literacy rate for Malay, which was 95.2% [36].

Measurement equivalence was demonstrated by the results of the psychometric analyses and comparison of the results with those of the German study [15]. Fit statistics showed that multidimensional structure of the constructs for this population was the same as that of the German study [15]. The instrument was also invariant across age groups, indicating that the latent variables have the same meaning for the population across ages. The internal consistencies of the subscales were satisfactorily within the recommended range of 0.70 and 0.95 [25] except for subscale AC where the Cronbach alpha coefficients were 0.52 for 12–14 year old age group, 0.62 for 15–17 year old age group and 0.56 for 12–17 year old age group. The relatively lower values of Cronbach alpha were expected because the AC subscale consisted of 2 items only and for a subscale with only a few items, Cronbach alpha coefficient of 0.5 is considered acceptable [37, 38].

The adapted English PIDAQ’s discriminant validity showed statistically significant differences in PIDAQ mean scores between those with slight and severe malocclusion based on self-rated malocclusion index (MI-S) for all subscales and age groups. Similar to the past study [15], the effect sizes of the differences were high with positive effect between the severity of malocclusion and the PIDAQ mean scores of the SI, PI and AC subscales, respectively, and negative effect between the severity of malocclusion and the DSC subscale mean scores. Similar results were observed when the malocclusion was rated by the investigators (MI-D) for the older (15–17 years old) and the overall (12–17 years old) age groups. When malocclusion was assessed by the investigators, the effect sizes were much lower than when assessed by the participants themselves. This pattern was also similar to that found by Klages et al. [15]. It shows that the same level of malocclusion may not be rated equally between patients and professionals. It is possible that the differences is due to the investigators assessing the malocclusion in an objective manner while the participants assessed their level of malocclusion based on self-perception of the oral impacts. This is a common observation to have patients complaining that their malocclusion is worse than that assessed by clinicians.

For the younger age group (12–14 years old), a statistically significant difference was only detected between the DSC mean scores of those who were rated by the investigators with slight or with severe malocclusion. The SI, PI and AC scores of those with slight malocclusion as rated by the investigators were lower than those rated by the investigators as having severe malocclusion. Although the trend was similar to that found in the past study [15], the differences were not statistically significant. The lack of statistical significance may be due to a higher number of investigators involved in this study, which might introduce some inconsistencies despite the calibration that was done prior to the study. However, considering that the PIDAQ mean scores of the self-rated malocclusion (MI-S) by the younger age group had very strong effect sizes and that the PIDAQ mean scores of the investigator-rated malocclusion (MI-D) by the overall age group were significant different, these have provided adequate evidence for the discriminant validity for the instrument.

Apart from discriminant validity, the study also included further evidence of the construct and criterion validity properties of this instrument by comparing the PIDAQ mean scores of participants’ self-assessed ranking of dental appearance, need for braces and impact on daily performances attributed to malocclusion scores (CS-OIDP). This was based on the argument that those with impact on their OHRQoL due to dental aesthetics would have lower ranking of self-perceived dental appearance, higher perception of the need to correct their dental alignment by braces and also some impact on their daily performances. It should be noted that in this study, the Child-OIDP instrument was based on Yusuf et al*.* [39]. Conceptually, the instrument has been found to be valid for young Malaysian adolescents [39]. During the pilot test, the participants understood the content of the instrument with no modifications required. This may be due to the high literacy rate of 93.2% for basic English among Malaysian adolescents, as found in a sample of 5000 secondary schoolchildren in Malaysia [36], and the language used for the instrument was not ambiguous to this population as it was geared to 10- to 11- year-old UK primary school children [29]. In terms of construct validity, regardless of age group, those with low DSC mean scores and high SI, PI, AC and total PIDAQ mean scores had lower perception on their dental appearance and felt that their dental alignment needed to be corrected by braces. In terms of criterion validity, those with high total PIDAQ scores also had impacts on their daily activities, which were attributed to malocclusion. This indicated that those with psychosocial impact due to the influence of dental aesthetics would also have their daily activities affected that was accounted by malocclusion.

In the reproducibility test analysis, the ICC scores for the DSC subscale for all age groups and the ICC scores for the SI subscale in the younger age group were excellent, with values well above the recommended minimum level of 0.70 [25]. The ICC scores for the remaining subscales in the rest of the age groups were in the range of fair to good [28]. The ICC scores of subscales in the older age group were slightly lower than those in the younger age group. A few factors may have contributed to the slightly reduced ICC scores than the recommended level. Firstly, although English is widely used as a lingua franca, the prevalence of its use among adolescents may vary. This study included participants from both urban and rural schools. Although the students were able to answer the Malaysian English version of the PIDAQ, the levels of proficiency may be slightly different between urban and rural schools as more emphasis in the use of English is often seen in urban schools. The inclusion of participants from rural schools, despite their ability to answer the English version of the PIDAQ, may have introduced some variations in interpretations that resulted in the less favourable ICC values in this study. Secondly, as the AC subscale has only 2 items and social impact perceptions of malocclusion may vary over time (assessed by SI subscale), very good reproducibility of these subscales cannot be expected at all times. To further support the reproducibility, the paired *t*-test demonstrated that the differences in mean scores between the first and second questionnaire administration were small and not statistically significant. As such, the relatively wider 95% CI values for ICC of AC and SI subscales in the older age group were deemed acceptable.

In terms of the floor and ceiling effects, the adapted English PIDAQ had neither significant floor effects nor ceiling effects. This suggests that the instrument was sensitive enough to discriminate those with the lowest and highest possible scores [25]. The results were better than the German PIDAQ, which demonstrated some floor effects in the SI, PI and AC domains [15].

## Conclusion

Following a few modifications in the cross-cultural adaptation of the English PIDAQ for Malaysian adolescents, the shortened version of the Malaysian English PIDAQ’s functional equivalence was established. The instrument is empirically valid and reliable to assess Malaysian adolescents’ OHRQoL specific to malocclusion.

### Symbols

α: alpha; ∆: Differences; *χ* ^2^ : Chi-square

## Abbreviations

AC: Aesthetic concern
ANOVA: Analysis of variance
CFI: Comparative fit index
Child-OIDP: Child oral impacts on daily performance
CS-OIDP: Condition-specific oral impacts on daily performance
DSC: Dental self confidence
ICC: Intraclass correlation coefficient
IOTN: Index of orthodontic treatment need
IOTN-AC: Aesthetic component of the index of orthodontic treatment need
IOTN-DHC: Dental health component of the index of orthodontic treatment need
MI-D: Malocclusion index (Investigator-rated malocclusion)
MI-S: Malocclusion index (Subject-rated malocclusion)
OHRQoL: Oral health related quality of life
PI: Psychological impact
PIDAQ: Psychosocial impact of dental aesthetics
POS: Perception of occlusion scale
RMSEA: Root mean square error of approximation
SDC: Smallest detectable change
SEM: Standard error of measurement
SI: Social impact
